# Foveated Retinotopy Improves Classification and Localization in Convolutional Neural Networks

**DOI:** 10.3390/vision10020017

**Published:** 2026-03-30

**Authors:** Jean-Nicolas Jérémie, Emmanuel Daucé, Laurent U. Perrinet

**Affiliations:** 1 Institut de Neurosciences de la Timone, Aix-Marseille Université, CNRS UMR 7289, 13005 Marseille, France; jean-nicolas.jeremie@univ-amu.fr (J.-N.J.);; 2 Ecole Centrale Méditerranée, Technopôle Château-Gombert, 13013 Marseille, France

**Keywords:** foveated vision, convolutional neural networks, transfer learning, visual categorisation, neuromorphic transformation, NeuroAI

## Abstract

From falcons spotting prey to humans recognizing faces, the ability to rapidly process visual information depends on a foveated retinal organization that provides high-acuity central vision while preserving low-resolution peripheral vision. This organization is conserved along early visual pathways, yet remains under-explored in machine learning. Here, we examine the impact of embedding a foveated retinotopic transformation as a preprocessing layer on convolutional neural networks (CNNs) for image classification. By applying a log-polar mapping to off-the-shelf models and retraining them, we achieve comparable accuracy while improving robustness to scale and rotation. We demonstrate that this architecture is highly sensitive to shifts in the fixation point and that this sensitivity provides an effective proxy for defining saliency maps that facilitate object localization. Our results demonstrate that foveated retinotopy encodes prior geometric knowledge, providing a solution for visual searches and a meaningful classification robustness and localization trade-off. These findings provides a proof of concept in order to connect principles of biological vision with artificial networks, suggesting new, robust and efficient approaches for computer vision systems.

## 1. Introduction

How can we design convolutional neural networks (CNNs) that are robust to geometric transformations (e.g., rotation, zoom) while also enabling precise object localization? This paper explores whether incorporating a biologically inspired foveated retinotopic transformation as a preprocessing layer can address this challenge.

### 1.1. Biological Inspiration: Foveated Retinotopy

For predators like birds of prey [1] or sharks [2], efficiently detecting prey is critical for survival. More broadly, visual search—the process by which organisms scan their environment to locate and identify objects of interest—is essential across species. This includes predators like falcons and sharks, as well as herbivores such as howler monkeys, fruit bats, hummingbirds, and parrots. Many of these species share a visual system characterized by foveated retinotopy [3], where the visual field is represented more densely around a central region. This organization is believed to underpin the efficiency of visual search.

Many species, including humans and mammals, possess a foveated retinotopy—a visual system with a high-acuity central region and lower-resolution peripheral regions [3,4]. This organization is characterized by a radial decrease in photoreceptor density with eccentricity, defining a *log-polar* mapping [4]. Functional magnetic resonance imaging (fMRI) studies have shown that this radial organization is maintained in early visual cortex (V1, V2, and V3), where different regions are activated depending on the position of stimuli in the visual field [5,6].

The evolutionary advantage of foveated retinotopy remains debated. It may facilitate efficient parallel processing of spatial features [7,8] or minimize global wiring length [9]. Alternatively, it could be an artifact of developmental scaffolding [10]. Some species, such as eagles and bottlenose dolphins, have evolved a dual fovea—one for lateral vision and one for forward vision, highlighting how retinal topography may adapt to a given ecological niches [2]. More broadly, this organization raises the question of how the visual system builds an egocentric model of local space from parcellated retinal input [11].

### 1.2. Eye Movements and the Sequential Analysis of the Visual Scene

Foveated retinotopy facilitates visual exploration by enabling efficient processing when the eye actively moves to focus on points of interest. This is achieved through oculomotor behaviors such as saccades (rapid eye movements) and fixations (periods of stable gaze). During visual exploration, saccades move the fovea to successive points of interest, while fixations allow precise processing of visual information. This alternation between saccades and fixations forms the basis of our scan path and influences how we perceive and interact with our environment [12]. This behaviour is guided by the Visual Search process.

The study of visual search in humans involves understanding the mechanisms controlling eye movements and visual attention. Eye movements are influenced by factors such as object saliency [13]. Pioneering work by Yarbus [14] and Noton and Stark [12] introduced the concept of a “scan path” as the trajectory of eye movements during visual exploration. Eye-tracking studies have since shown that our eyes follow predictable patterns that maximize the efficiency of visual information acquisition [15]. Recent studies further demonstrate that the combination of saccades, fixations, and foveal input enhances visual acuity and supports the integration of local features into global perceptual representations [16,17,18].

### 1.3. The Log-Polar Model in Computer Vision

The log-polar model, inspired by biological foveated retinotopy, has become a cornerstone in artificial vision. Early work by Sandini and Tagliasco [19] demonstrated how sampling the visual scene as a function of eccentricity can compress visual information, emphasizing the center while shrinking the periphery. The log-polar model, formalized by Araujo and Dias [20], represents the visual field using polar coordinates: azimuth (angle) and eccentricity (distance from the center) on a logarithmic scale. This results in a resolution that decays exponentially with distance from the center [20]. Recent advances in neuromorphic computing have enabled real-time implementation of this transformation [21].

**Figure 1:**
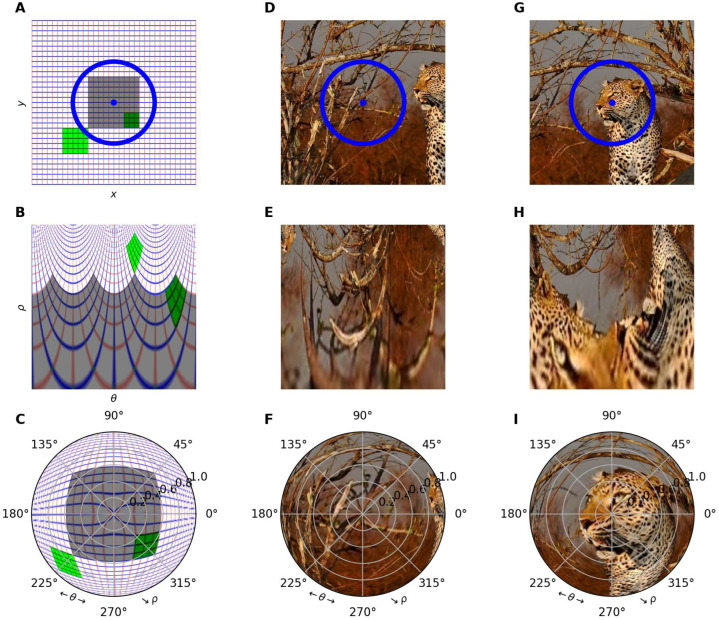
We illustrate the process of mapping input images defined in Cartesian coordinates to a foveated retinotopic space using a log-polar transformation. The fixation point is marked by a blue disk and the approximate area of the fovea by a blue circle. In (**A**), the input image is defined as a regular grid representing the Cartesian coordinates (*x*, *y*) by vertical (red) and horizontal (blue) lines. As shown in (**B**), by applying the log-polar transform to this image, the coordinates of each pixel with respect to the fixation point are transformed based on its azimuth angle θ (abscissa) and the logarithm of its eccentricity $\rho =\log(\sqrt{x^{2}+y^{2}})$ (ordinates). This transformation results in a fine-grained representation of the central area and a deformation of the visual space. Note that the green square is translated in retinotopic space when it is scaled and rotated. The third row (**C**,**F**,**I**) illustrates the reconstruction of the image in question, exhibiting a over-representation around the point of fixation in the Cartesian reference frame. When the transformation is applied to a natural image, as shown in (**D**,**G**), there is a noticeable compression of information in the periphery in the log-polar referential (see (**E**,**F**,**H**,**I**)). Also, this representation is highly dependent on the fixation point, as indicated by the shift shown in (**G**–**I**) when the fixation point is moved to the right and up.

The log-polar transformation introduces geometric distortions, enlarging central objects and shrinking peripheral ones (Figure 1). Critically, it converts rotations and zooms into translations in log-polar space: rotations affect the azimuth angle θ, while zooms influence the logarithmic eccentricity ρ. This property makes the log-polar model uniquely suited for handling geometric transformations, as rotations and zooms in Cartesian space become simple shifts in log-polar space (Figure 1A,D,G).

The log-polar model has been widely applied in computer vision, particularly for template matching [20,22,23,24] and robotics [25,26]. However, its low peripheral resolution poses challenges for information retrieval, as it is difficult to predict which regions contain the most relevant information before eye movements occur. Approaches to address this include bottom-up methods, which rely on low-level features [13], and top-down methods, which use prior knowledge to guide attention [27]. Recent studies have further explored foveal retinotopy, showing that incorporating log-polar references can improve rotation invariance in CNNs [28,29,30].

### 1.4. Convolutional Neural Networks and Translational Invariance

Convolutional neural networks (CNNs) have achieved remarkable success in visual recognition tasks, such as the ImageNet challenge [31], often surpassing human accuracy [32]. CNNs are well-suited for image processing due to their factorized operations, adaptive convolutional kernels, and translation invariance achieved through pooling layers [33]. While CNNs are not biologically plausible [34], their properties—such as parallel processing and increasing receptive field sizes—resemble aspects of biological vision.

Despite their success, CNNs remain vulnerable to adversarial attacks, where imperceptible modifications can cause misclassification with high confidence [35,36]. This vulnerability limits their applicability in safety-critical domains, necessitating more robust models. Insights from biological neuroscience may be critical to achieving this goal [37].

The contribution of foveated retinotopic mapping to visual processing in CNNs remains underexplored. The log-polar transformation could act as a “deep prior” to constrain the range of achievable representations, providing a more efficient way to learn the structure of the visual world [38]. Prior work has shown that transformations like log-polar [39], polar coordinate mapping [40], and spherical transformations [41] can enhance CNN robustness, particularly to rotations.

### 1.5. Paper Contributions

This paper investigates the benefits of incorporating foveated retinotopy into CNNs for image classification tasks. By aligning computational models with neurobiological principles, we aim to enhance the performance of artificial systems and contribute more biologically plausible models to the NeuroAI community.

Our key contributions are:

- Demonstrating that conventional CNNs are vulnerable to geometric transformations (e.g., rotations, zooms), highlighting a fundamental limitation in current architectures.
- Introducing a biologically inspired foveated layer using a log-polar transformation at the network input, which provides rotation and scale invariance to the convolution layers.
- Evaluating our approach on standard off-the-shelf architectures and benchmarks, and quantifying the impact of foveated vision on classification performance.
- Showing how the foveated input layer enhances CNN robustness to geometric transformations and provides insights into the relationship between network architecture and invariance properties.
- Demonstrating that analyzing the network output in probabilistic terms reveals class-likelihood variations across multiple viewpoints, enabling effective object localization without additional training.
- Establishing deeper connections between computational and biological vision, paving the way for improvements in machine learning architectures and a better understanding of natural visual processing.

## 2. Methods and Materials

### 2.1. The Log-Polar Transform

In most mammals and amphibians, the arrangement of the (external) visual field is preserved in the early visual pathway, a feature called retinotopy. Retinotopic mapping results from the combined effect of the arrangement of photoreceptors in the retina and their output convergence via the optic nerve. This causes nearby regions of the visual field to activate adjacent neural structures as signals travel from the retina to the brain. These mappings differ from species to species, and our study concentrates on foveated vision (as in humans) which gives more resolution to the central field of view. In particular, we implement it by transforming the Cartesian pixel coordinates into log-polar coordinates [25].

This simple parameterized transformation models accurately this biologically inspired retinotopic mapping. Considering arbitrary images (potentially with multiple channels such as RGB), each pixel’s position is defined by two real coordinates (x,y) on a Cartesian reference frame. By convention, *x* and *y* are here considered belonging to the interval [−1,1], with (0,0) being the center of the image. To implement the concentration of pixels near the center of the retina, we need to consider an *irregular* grid in the Cartesian referential that maps to a *regular* grid in the log-polar referential. In the log-polar referential, the location of each pixel has corresponding coordinates (ρ,θ) as defined by previous studies [42] by:

(1) $$\begin{array}{ccc} \rho & = & \log_{2}\sqrt{{\left(x-x_{0}\right)}^{2}+{\left(y-y_{0}\right)}^{2}} \end{array}$$

(2) $$\begin{array}{ccc} \theta & = & \arctan(\frac{y-y_{0}}{x-x_{0}}) \end{array}$$

with $\left(x_{0},y_{0}\right)$ defining the “center of fixation” (see Figure 1) [43]. Importantly, ρ and θ are only defined for $\left(x,y\right)\neq \left(x_{0},y_{0}\right)$. In most of our experiments, we consider $x_{0}=\left(0,0\right)$, allowing us to focus on the central part of the image. Each dot (ρ, θ) in the log-polar coordinate system has thus a unique correspondence in the Cartesian coordinate system (and vice versa): For each (logρ, θ) belonging to the grid, the corresponding pixel coordinate is $\left(x_{0}+\rho \cos\theta ,y_{0}+\rho \sin\theta \right)$.

In practice, images have a finite resolution and to avoid biases in the evaluation between networks, the number of angles sampled ($N_{\theta }$) and the number of eccentricities sampled ($N_{\rho }$) are set to 224, so that the size of the transformed image match the resolution 224×224 of the input images. Note that native image resolution is generally higher than that used during network processing, with the average resolution in the Imagenet dataset being around 500×500 pixels. This transformation is performed with the PyTorch library [44] through the use of the gridsample() function, which maps the pixels of an input image to the coordinates of any arbitrary grid, using a linear interpolation to estimate the value of the pixels. This function is used, for instance, in spatial transformer networks [45].

Let’s now define each coordinates. All θ values are within a linear distribution in [0;2π), while ρ values are within a logarithmic interval with $r_{\min}$ the minimal radius and $r_{\max}$ be the maximal radius (with $N_{\rho }$ the radial resolution). In practice, we use a log-polar grid with an outer log-radius of $\log_{2}r_{\max}=0$ ($r_{\max}=1$, defining a circle tangent to the image box) and an inner log-radius of $\log_{2}r_{\min}=-5$ ($r_{\min}=2^{-5}$). In summary, the regular grid is the interval $\left[\log_{2}r_{\min},\ldots ,\log_{2}\left(r_{\min}+i\times \frac{r_{\max}-r_{\min}}{N_{\rho }-1}\right),\ldots ,\log_{2}r_{\max}\right]$, for *i* in $\left[0,\ldots ,N_{\rho }-1\right]$, in the $\log_{2}\rho$ dimension and $\left[0,\ldots ,j\times \frac{2\pi }{N_{\theta }},\ldots ,2\pi \left(1-\frac{1}{N_{\theta }}\right)\right]$, for *j* in $\left[0,\ldots ,N_{\theta }-1\right]$ in the θ dimension. Note that some pixels in the log-polar grid may be smaller than the pixels from the Cartesian grid.

### 2.2. Convolutional Neuronal Networks (CNNs)

Convolutional neural networks (CNNs) have become essential tools in image classification, with several pre-trained models available for download. For example, the VGG family, including VGG16 and VGG19, introduced by Simonyan and Zisserman [46], uses deep architectures with 16 or 19 layers, consisting of stacked convolutional layers followed by fully connected layers. ResNet (Residual Networks), introduced by He et al. [32], addresses the vanishing gradient problem in deep networks by incorporating skip connections, allowing for the training of extremely deep networks (e.g., ResNet 50, ResNet 101 with respectively 50 and 101 layers). These models are widely used due to their robustness and scalability. The implementation of these deeper networks have demonstrated that deeper networks display enhanced resilience; however, this improvement is coupled with an overall increase in computational complexity [32]. Therefore, based on these findings, we focus on the deep CNN ResNet (with 18 to 101 layers from the PyTorch library on the ImageNet-1K [31] categorization challenge which consists in classifying natural images into 1000 labels. We further introduced a circular padding in the convolutions, however we controlled this had little impact overall.

### 2.3. Datasets

Typical image classification datasets in machine learning consist of sets of RGB images at varying resolutions, each associated with a unique label. The classification task involves learning a parametric function that maps pixel representations of the image to a label. This example-based learning assumes the existence of separable regions within the representation (or feature) space, enabling the model to distinguish between classes irrespective of variations in position, size, orientation, lighting, or contrast.

**Figure 2:**
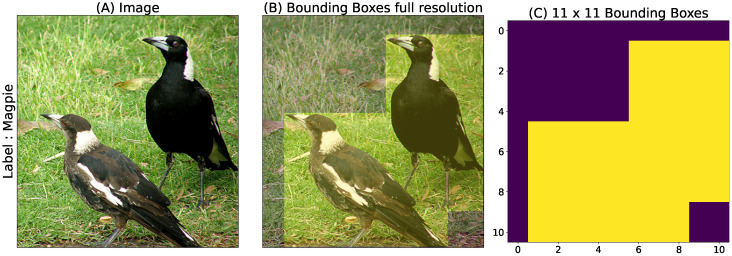
(**A**) Original image from the ImageNet dataset. (**B**) Bounding-box annotation shown as a heat map. (**C**) Downsampled heat map (11×11) used as ground truth for localization. The “regular” dataset uses the full image as input (Cartesian or log-polar for the retinotopic framework), whereas the “focus” dataset uses the cropped bounding box of the target label (yellow boxes in **B**). To ensure consistency, if there are multiple boxes present, only the first annotation provided in the dataset is used when constructing the “focus” dataset.

Two datasets were used for our study: the first dataset is the one from the ImageNet [31] challenge, which is the most widely benchmarked due to its huge collection of images and associated labels (the subset of ImageNet used in this study, i.e., ImageNet-1K with 1000 labels and about 1000 examples per label). In addition to the classification task, we consider here the localization task, which is prominent in computer vision. It consists in identifying both the label and the position of one or more objects of interest in an image. A distinction can be made between (i) the *visual search* task, where the label is given in advance and the task is simply to find the position of the object in the image, and (ii) the *image labeling* task, which consists in identifying both the objects and their position in the image, in the form of a labeled bounding box (see Figure 2). Despite its advantages, ImageNet has limitations for localization tasks. For example, the dataset lacks multi-labeling, with only one label per image. It is worth mentioning that ImageNet has some biases, the objects of interest are generally centered in the images, and the proportion of bounding boxes relative to the image size is relatively large, which may limit the impact of certain analyses.

We consider two dataset configurations for the ImageNet dataset: In our first configuration, the center of gaze is set to the center of the original image, taking advantage of the fact that most ImageNet images are human-made and that photographers have a bias toward centering the object of interest. This defines our “regular” image dataset. Notwithstanding the above, this a priori assumption of centered position is not sufficient to generate a dataset perfectly suited for retinotopic transformation. In a second setup (called the “focus” dataset), we use the bounding box information provided by the ImageNet dataset. Consequently, a sample is selected, defined as the smallest square containing a bounding box, assuming a center of gaze now at the center of the bounding box for the label of interest. This novel dataset is used to train a second generation of networks. Again, we use a circular mask for the Cartesian frame. This approach is more robust to the position of the visual object, but requires reliable bounding boxes.

**Figure 3:**
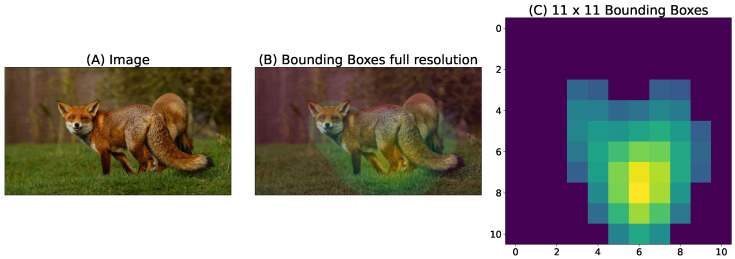
(**A**) Original image of the Animal 10k dataset. (**B**) Heat map generated by fitting Gaussians to annotated key points. (**C**) Heat map from (**B**) normalized and downsampled to 11×11, used as ground truth. A threshold of 0.2 is applied to delineate the assumed animal contour.

Moreover we also used the Animal 10k dataset, as referenced by Yu et al. [47], with the goal of identifying the animals depicted in these images. This dataset contains 10,015 images, each depicting an animal. The dataset also provides a set of key points for each animal present in an image. For each image from Animal 10k containing a set of keypoints, we created a Gaussian heatmap centered on those points, with the peak value set to 1 and values decreasing with a standard deviation proportional to object size, thus capturing the true spatial extent and location of the target animal within each image (see Figure 3). This approach allows for more effective localization and analysis of the visual distribution of animals in images.

### 2.4. Datasets Transformations and Transfer Learning

In this study, three series of transformations were used, depending on whether the network used the Cartesian or the retinotopic reference frame. In the case of the latter, a log-polar grid is used to facilitate the transformation of the image into the retinotopic frame. Due to the intrinsic nature of the transformation, which results in the cropping of a circular sample within the original image, a circular crop is implemented for the Cartesian frame to ensure comparability. To allow for a more straightforward comparison, the “raw” datasets were processed without a circular mask or polar logarithmic transformation. Each set underwent a uniform transformation, including normalization to tensors and resizing to a resolution of 224×224 to match the pre-trained parameters of the model.

To assess the efficacy of our retinotopic mapping, we examine popular off-the-shelf CNNs pre-trained on standard, large image datasets. These networks are re-trained on our datasets, either with (or without) a log-polar retinotopic transformation, using the cross-entropy loss from the PyTorch library. We use the stochastic gradient descent (SGD) optimizer from the PyTorch library and validate parameters such as batch size, learning rate, and momentum by performing a sweep of these parameters for each network. During the sweep, we vary each of these parameters over a given range while leaving the others at their default values for 1 epoch on 10% of the entire ImageNet training dataset. We choose the parameter values that give the best average accuracy on the validation set: batch size = 80, learning rate = 0.001, momentum = 0.9.

We retrained the networks for 10 epochs using the full training dataset, maintaining identical learning parameters throughout. To ensure robustness, we monitored validation accuracy and confirmed that stable performance was achieved within 2 epochs. All reported accuracies reflect this early convergence.

### 2.5. Attacking Classical CNNs with a Geometrical Rotation

A common approach to evaluating the robustness of deep learning models is to subject them to adversarial attacks. In this study, we investigate the robustness of the deep learning models to natural image transformations that are easily perceived by humans. In particular, we evaluated the performance of the networks on the ImageNet dataset when the images were rotated by different angles and averaged the accuracy for each angle. To further evaluate the robustness to rotations, we also designed a “rotation-based attack” scenario. To perform such an attack on a model *m*, we follow this simple procedure. Given an image *I* and the output of the model p=m(I), which returns a probability vector over K=1000 classes, the loss function L is defined as the cross-entropy between the predicted probability vector and the ground truth label *y*, which we denote as L(m(I),y). This is the loss minimized during gradient descent training. We then systematically rotated the images and tracked the change in model loss. By denoting a rotation of the image by an angle θ as rot(I;θ), we define the rotation-based attack as the following heuristic for each image in the dataset:

(3) $$\begin{array}{ccc} \bar{\theta } & = & \underset{\theta }{arg max}L\left(m\left(\mathrm{rot}\left(I;\theta \right)\right),y\right) \end{array}$$

(4) $$\begin{array}{ccc} \hat{y} & = & \underset{k}{arg max}\left({\bar{p}}_{k}\right)\;\mathrm{with}\;\bar{p}=m\left(\mathrm{rot}\left(I;\bar{\theta }\right)\right) \end{array}$$

More specifically, our approach is to first choose the rotation angle that maximizes the loss, and then infer the most likely label for that particular angle. As a result, we can compute the concordance between the predicted label $\hat{y}$ for the image rotated at the angle $\bar{\theta }$ with the worst loss with respect to the ground truth label *y*. Using this procedure, we calculated the overall accuracy on the entire test set, quantifying the network’s brittleness to natural image rotations. We use a similar strategy for other geometric transformations, such as zooms or translations.

### 2.6. Localization Tools and Evaluation

A widely accepted technique for evaluating the performance of Convolutional Neural Networks (CNNs) in localization tasks is the Class Activation Mapping (CAM) method. CAM works by analyzing the output of the CNN with respect to the target class, assigning weights to activations in each spatial feature map. This process generates a heat map that highlights significant areas of the image based on their contribution to the prediction. Building on the foundation of CAM, several derivative methods have emerged, including Grad-CAM [48], Score-CAM [49], and Opti-CAM [50].

In an effort to fairly quantify the respective contributions of each method, many quantification techniques have been developed. Here we select some of them to compare the models using the retinotopic or Cartesian reference. Energy-Based Pointing Game: Localization is successful if the peak activation of the heatmap of a given label is inside the ground true mask (or box). Mean Activation IN: Mean activation of the heatmap of a given label inside the ground true mask. Mean Activation OUT: Mean activation of the heatmap of a given label outside the Ground True mask. Mean Activation Ratio: Ratio of activation inside and outside the box; the higher the value, the more efficient the heatmap is at indicating the position for a given label. Intersection over Union (IoU): Ratio of the area of overlap between the heatmap and the ground truth to the area of union between the heatmap and the ground truth. Peak-IoU and Peak-Threshold: For a modulation of a threshold on the heat map, the Peak-IoU is the maximum IoU value reached at the Peak-Threshold.

### 2.7. Visual Object Localization: Protocol

Deep convolutional networks such as ResNet output a vector of real numbers which predict the logit of the likelihood in label space, and this prediction is optimized through cross-entropy loss. Applying the softmax function allows the output to be interpreted as a probability vector, assigning a probability of presence to each of the 1000 labels (likelihood score).

This allows us to make a binary decision (“present” or “not present”), e.g., by selecting the label corresponding to the highest likelihood. In our setting, we can also take different views of a large image and compute the likelihood for each of them, allowing us to compare which view provides the best likelihood. Views can consist, for example, of focusing on regions of the image centered on different fixation points, with the fixation points aligned on a regular grid in visual space.

**Figure 4:**
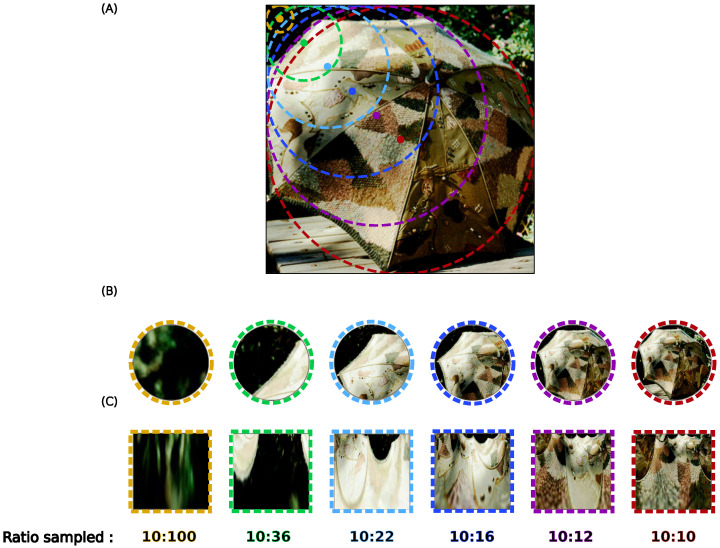
We illustrate the protocol used to obtain the likelihood map with an example on a single image (**B**). We show one sample for each sample ratio size (1:10 to 1:1, from left to right). (**A**) The samples are cropped on the image in the Cartesian frame, we use circular cropping to match the area covered by the log-polar frame. (**C**) The corresponding samples cropped on the image in the retinotopic frame.

We used two parameters to define these maps. The first parameter is the resolution of the fixation point grid, and when not specified this resolution is set to 11×11. The second is the size of the samples clipped at each of these positions, defined as the ratio of the log-polar grid radius of the input to the total input size or Cartesian grid size, since the grid is a square for Cartesian samples (for an illustration of this simple process, see Figure 4). At each viewpoint, the largest possible sample is cropped. Thus a minimum sample with 1:10 ratio at the border and the whole image at the center. From the Cartesian or retinotopic reference frame, this sample is then resized, if necessary, to a 224×224 resolution to match the input size for the CNN before processing or transformed by the retinotopic mapping (also at 224×224 resolution) before being used as input for the corresponding network, see Figure 4B,D. Conveniently, a collection of samples for different fixation points can be processed as a single batch. This protocol defines a likelihood map for any given network as the likelihood of categorizing the presence of a label of interest inferred at regularly spaced fixation points in the image.

## 3. Results

### 3.1. Training on Transformed Images

We retrain pre-trained ResNet [32] networks on different variants of the ImageNet-1K dataset [31] (see Section 2.3), either using a simple circular mask applied on the raw images (hereafter called the Cartesian dataset) or using log-polar transformed images (hereafter called the “retinotopic” dataset). Two variants of the training sets are also considered. In a first case (called the “regular” case), the mask or the log-polar transformed images are applied to the “regular” images. In a second case (called the “focus” case), the mask or the log-polar transformation is focused on the center of the *bounding boxes* surrounding the objects of interest and which are provided with the data set. The pre-trained networks are first re-trained on the “regular” version of the dataset, generating a first generation of networks, and then a second generation is trained by fine-tuning these networks on the “focus” dataset (see Figure 2). We then compare the classification accuracy of the original and the different re-trained networks. In this section we choose to express the accuracy as the percentage of correct categorization on the corresponding validation dataset.

The baseline classification accuracy of the standard pre-trained ResNet network, which we will call the “raw” network (as it has no mask, retinotopic transformation nor training), averaged on the Cartesian test dataset is 81.7%. In comparison, our re-trained networks show respectively accuracies of 78.5% and 74.3% on the “regular” Cartesian and retinotopic datasets, and 82.5% and 77.4% on the “focus” Cartesian and retinotopic datasets. As we can observe, when fine-tuning the networks on the “focus” dataset, despite reducing the image resolution by cropping the image, the accuracy is improved by 4.0% and 3.1% respectively. This demonstrates that this cropping tends to suppress distractors in the periphery and enhances average accuracy.

**Figure 5:**
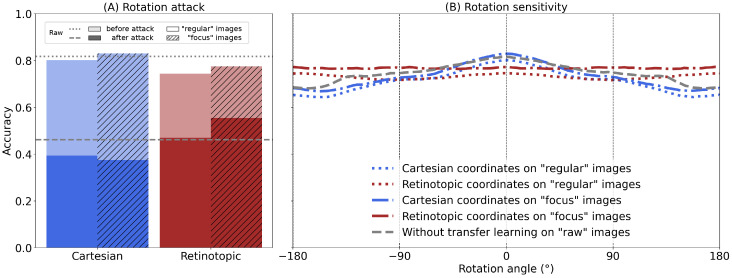
(**A**) For all the networks, we plot the accuracy averaged over the dataset without rotation (in light color), or for each image rotated at the angle $\bar{\theta }$ with the worst loss (rotation-based attack, in full color). No shading: regular dataset. Diagonal shading: focus dataset. Gray dashed lines: accuracy of the “raw” network (without any transformation nor training). (**B**) The average accuracy is shown for both Cartesian or retinotopic re-trained networks, and the “raw” network, with different image rotations. The rotation is applied around the central fixation point with an angle ranging from $-180^{{}^{\circ}}$ to $+180^{{}^{\circ}}$ (in steps of $15^{{}^{\circ}}$).

**Figure 6:**
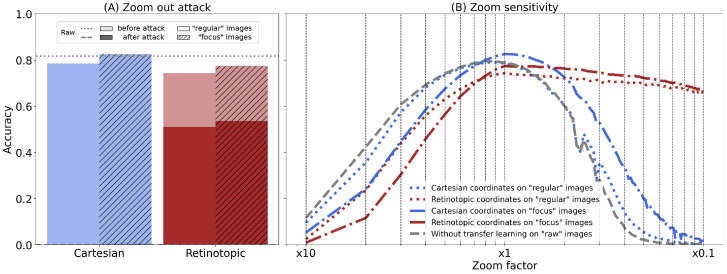
(**A**) For all networks, we plot the accuracy averaged over the unzoomed dataset (in light color), or for each image zoomed out to the scale with the worst loss (zoom out attack, in full color). The gray dashed lines represent the accuracy of the “raw” network (without re-training). (**B**) Average accuracy over a sample of 50,000 images from the ImageNet validation dataset, shown for both re-trained and pre-trained networks with different input image zooms. The zoom is applied at the central fixation point with a zoom factor geometrically-spaced from ×10 to ×0.1.

Furthermore, our networks re-trained on the log-polar transformed images have a slightly lower categorization accuracy than those re-trained on Cartesian images (seas will be shown in Figure 5A and Figure 6A). This result was expected because the log-polar transformation discards fine-grained information in the periphery without increasing central resolution. In fact, this rather limited loss of accuracy is quite remarkable for such a massive loss of information. One reason could be a general “photographer’s bias” for the “regular” images that tends to place the main object in the central region of the image. It is confirmed by the increase of accuracy observed when centering the point of fixation in the “focus” dataset.

### 3.2. Robustness of CNNs

To what extent can these re-trained networks be relied upon in practical applications? Indeed, a persistent challenge for deep neural networks is their lack of reliability in adversarial situations. For example, it is well documented that a minor alteration to the inputs of these networks, if well-designed, may result in a significant decline in classification accuracy. In particular, a classical robustness test applied to deep networks, called an “adversarial attack,” consists of perturbing independently the pixels in each image in order to maximize the error rate in the test phase (see Section 2.5). However, these modifications are typically subtle and perceptually resemble identically distributed independent noise, rendering them unlikely to happen in natural conditions. In contrast, biological agents interacting with their environment undergo significant visual perturbations, including large scene pans and tilts due to head and body movements.

As real-world objects appear in different orientations, we assess the resilience of our re-trained networks to geometric alterations of their inputs e.g., rotations and zooms. In comparison to modifying individual pixels, a rotation or a zoom represents a coherent, whole-image transformation controlled by a single parameter, namely the rotation angle or scaling factor. We thus choose to investigate for each network a simple attack scenario that maximizes the loss for different rotation angles for each image individually and then evaluates the classification result for this “worst” angle. Then, we compute the accuracy averaged over a sample of 50,000 images from the ImageNet validation dataset.

Our experiments show that while the original ResNet 101 achieves a nominal baseline accuracy of 81.7% on unperturbed images, a rotation attack significantly reduces the performance of the model. Applying the maximally deceptive rotation to each image reduces the average accuracy to 46.1% (see gray lines in Figure 5A). This is also true for our Cartesian network, with accuracy dropping to 39.3% for the Cartesian network re-trained on “regular” images, and even to 37.4% for the network fine-tuned on the “focus” dataset. In contrast, the retinotopic networks show a lower sensitivity to rotation attacks, with an accuracy reduced to 47.0% when using the “regular” images, and only to 55.4% when using the “focus” dataset.

This difference between the two types of networks is even more manifest in Figure 5B, where we compute the average accuracy over the test dataset for each single rotation angle. We first observe that the Cartesian networks show a decrease in accuracy with respect to the angle of rotation (with a symmetry with respect to horizontal flips), with a significant drop around $160^{{}^{\circ}}$, “raw” and “focus” accuracies were degraded to 66.8% and 68.1%, i.e., a drop of 16.1% and 13.4% (64.3% in “regular”, i.e., a drop of 15.8%). Strikingly, this effect is nearly absent in our retinotopic networks (see Figure 5B), which show a flat (invariant) accuracy over the whole range of rotation angles, with a minimal degraded accuracy at 76.4%, i.e., a drop of 0.8% (71.6% in “regular”, i.e., a drop of 2.9%). This marked difference can be interpreted as a consequence of the horizontal translation invariance found in classical CNNs. When applied to the retinotopic input space, this invariance transforms seamlessly into rotation invariance in visual space [20] (see [42] for a proof). Note that for all networks, no maximum accuracy exceeds the value obtained without rotation (i.e., $0^{{}^{\circ}}$ rotation angle) by more than 0.01%.

Analogous to rotation, zooming in and out is equivalent to a translation in log-polar space, and this property is expected to induce a similar invariance in the retinotopic networks. Similar experiments were therefore performed to test the effect of a zoom (see Figure 6), with a zoom ranging from ×10 to ×0.1, divided into “zoom-in” (×10 to ×1 range) and “zoom-out” (×1 to ×0.1 range). In Figure 6A, we applied the zoom attack only for the zoom-out case, i.e., ratios between ×1 and ×0.1, because zoom-in attack scenarios always lead to the maximum zoom-in, where the accuracy approaches zero. The response of all Cartesian networks (i.e., “raw”, “regular” or “focus”) reach chance-level performance when submitted to a zoom-out attack. Only the retinotopic networks keep a discriminative capacity in this case (51.0% on “regular” images and 53.6% on “focus” images), illustrating the importance of foveal information in categorization, especially when the peripheral information is scarce or deceptive.

Figure 6B illustrates the average accuracy across the entire zoom range, offering a comprehensive perspective on the effects of zooming in and out across our different networks. Unlike the previous case, a pronounced asymmetry emerges in both scenarios. Specifically, the various Cartesian networks tested (both the original and re-trained versions) exhibit an approximate symmetry relative to the logarithmic scale of zoom-in and zoom-out factors. In contrast, the retinotopic network displays an asymmetric response, characterized by a slightly declining plateau in the zoom-out direction and a more pronounced accuracy loss in the zoom-in direction compared to the Cartesian networks. When zooming in, both the retinotopic and Cartesian networks exhibit heightened sensitivity, with mean accuracy dropping near chance levels at a ×10 zoom. Notably, the Cartesian networks achieve marginally higher accuracies in this scenario, suggesting a potential architectural advantage under extreme zoom-in conditions. However, this finding should be interpreted cautiously, as it likely stems from the loss of visual detail and the blurring effect inherent to applying extreme zoom levels on low-resolution original images.

In contrast to the case of rotations, our fine-tuning on the “focus” dataset has a nuanced effect, as evidenced by its impact on the zoom optimal point. For the Cartesian network, the zoom corresponding to maximum accuracy is around ×2 in both its pre-trained and re-trained states. However, fine-tuning on the “focus” dataset shifts this optimal zoom to ×1. In comparison, the optimal zoom for the retinotopic network remains consistently at ×1, regardless of evaluation on “regular” or “focus” data. From an ecological perspective, zoom-out invariance is likely one of the most advantageous features in natural vision. This capability facilitates the detection of predators or prey at a distance, even in complex and cluttered environments where survival-critical information may appear at varying sizes. While this feature enhances the interpretation of visual scenes, it comes with a trade-off: the necessity to position objects of interest centrally on the retina.

**Figure 7:**
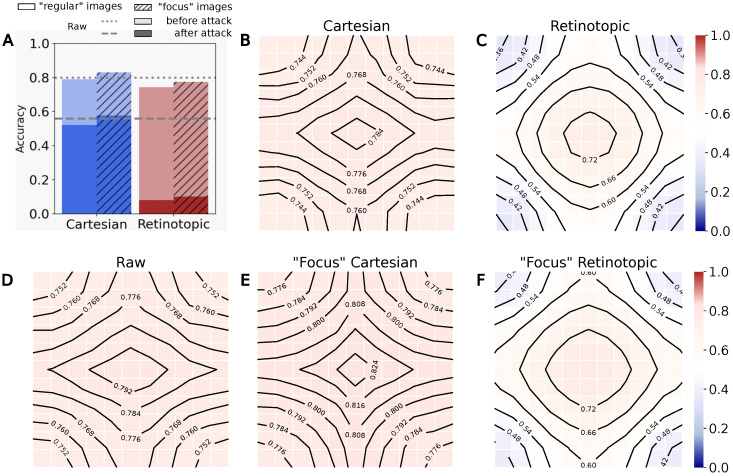
(**A**) For all networks, we plot the accuracy averaged over the validation dataset (in light color), or for each image translated (rolled) to the position with the worst loss (translation attack, in full color). The gray dashed lines represent the accuracy of the “Raw” network (without re-training). (**B**–**F**) Average accuracy over a sample of 50,000 images from the ImageNet validation dataset, shown for both re-trained and pre-trained networks (“Raw”, “Cartesian” or “Retinotopic”) with 7 different input images (regular or “Focus”) over different translations. Each translation is applied from the central fixation point and defines a linear grid of 11×11 points of fixation distributed regularly on the image.

Finally, based on these observations and the fact that translations in Cartesian space induce a significant, nonlinear transformation in retinotopic space (see Figure 1A,B), we investigate the effect of translations in Cartesian space on retinotopic networks. We thus investigate the effect of a rigid full-field translation by applying a roll function to the input image and place the fixation point at different positions in the image. Specifically, the fixation points are linearly distributed on an 11×11 grid. We then plot the mean accuracy when systematically selecting the worst position (based on the minimum loss as in the scenarios for a rotation Figure 5A or zoom-out attack Figure 6A) for the target label (see Figure 7A), or the mean accuracy of the networks as a function of the position repositioned in the center (see Figure 7B).

During this attack (see Figure 7A), the accuracy of the retinotopic networks is degraded to 10.1%, i.e., a drop of 67.3% (8% in “regular”, i.e., a drop of 66.3%), while the Cartesian “raw” and “focus” accuracies were degraded to 55.9% and 57.5%, i.e., a drop of 24% and 25.5% (52% in “regular”, i.e., a drop of 26.9%). As expected, in contrast to previous attacks, the resilience of retinotopic networks is below that of Cartesian networks. Looking at the average accuracy maps (see Figure 7B), we can see that the fixation points around the centre of the map have higher accuracy values than the fixation points around the periphery. This could be an artefact due to the photographer’s bias explained earlier. However, this effect is not observed on the Cartesian maps, which shows similar accuracy for all positions examined. Indeed, the robustness of our retinotopic networks to zoom-out and rotation comes at the cost of a high sensitivity to image translations. This increased sensitivity, although detrimental for classification tasks, is associated, as we will see, with an important capability to localize objects of interest in visual space, providing a basis for spatial processing in the brain.

### 3.3. Visual Object Localization: Likelihood Maps

**Figure 8:**
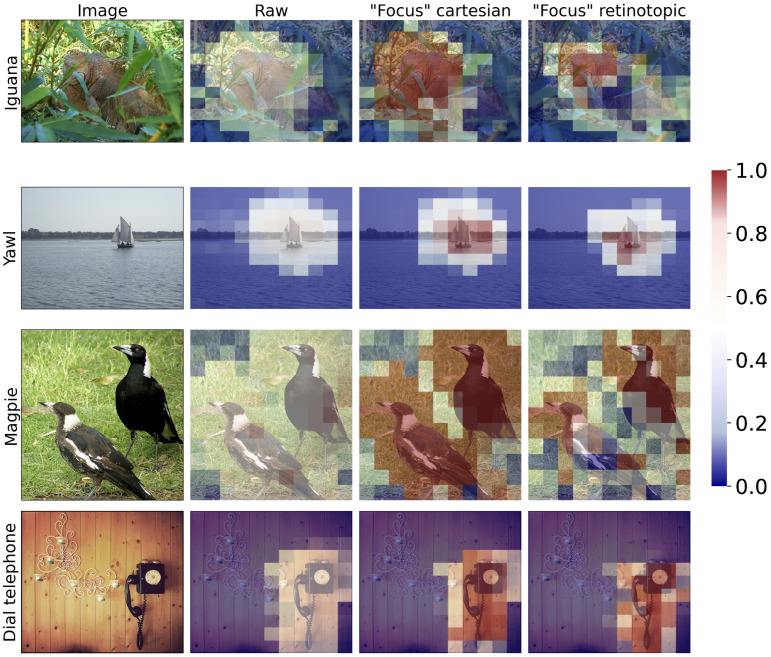
Likelihood maps computed on some prototypical images using 11×11 fixation points with the “raw” network, that is the original ResNet classifier with no re-training (second column), the network re-trained on images with circular mask on the “focus” dataset (third column), and the one re-trained on log-polar tranformed inputs (last column). The map displays the likelihood for the label of interest in the image.

To quantify the contribution of this sensitivity to translation, we consider here a new task, i.e., the *visual search* task, in which a visual object (of which the label is known in advance) needs to be localized over the entire image. Is is for instance known that such task allows for the quick retrieval of an image label [51]. We design a protocol for each network to allow us to compare different visual shifts, each one corresponding to a potential fixation point, and to generate a map of the expected (or actual) accuracy as a function of the fixation point (see Section 2.7). Let’s recall that in this protocol, a set of 11×11 fixation points is defined (with the coordinate (5,5) being the center of the image), and at each coordinate of the grid, a *likelihood* value is computed for the label of interest (see Figure 4). In practice, a likelihood is given at each location from a softmax calculation over the different labels, as in the classical ResNet classifier, providing a value between 0 and 1 for the label of interest. This projects the network output onto a 11×11 Bernoulli probability space corresponding to the likelihood of detecting the given label at each position, finally providing a “heat map” on our 11×11 grid in a way that is compatible with other localization protocols. We tested our likelihood protocol on the “regular” validation dataset (see Figure 8 for some examples).

#### 3.3.1. Mean Likelihood Maps

**Figure 9:**
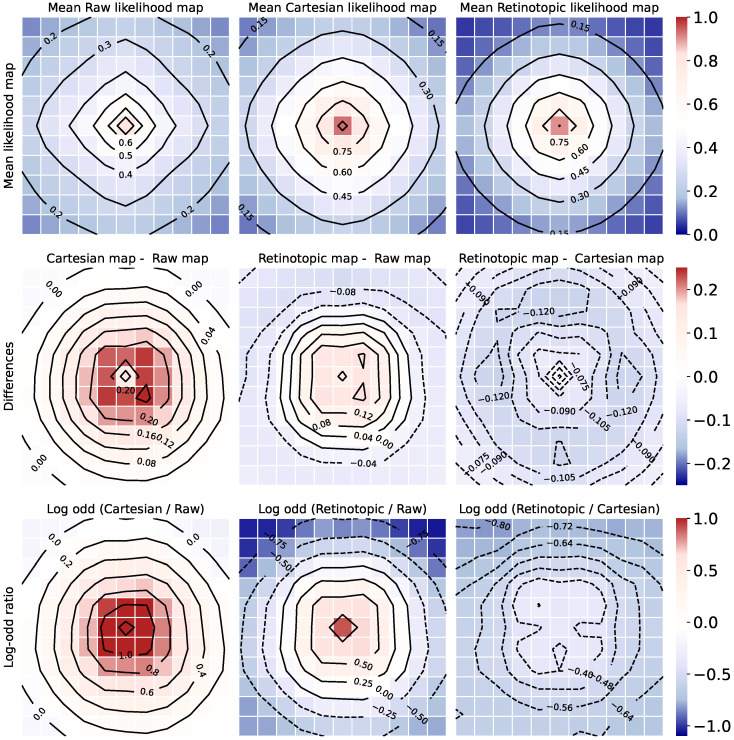
Mean likelihood on ImageNet’s validation dataset (50,000 images). From left to right: “raw” network (no re-training), Cartesian network retrained on the “focus” dataset, and Retinotopic network retrained on the “focus” dataset. **First row:** recentered maps, averaged over the validation dataset. **Middle row:** Difference maps. **Bottom row:** Log-odd ratio maps.

In Figure 9, we calculated the likelihood maps for all the images of the validation set, and re-centered them to place the viewpoint with the highest likelihood at the center of the grid. During the re-centering process, the spots outside the grid were assigned a Nan value to facilitate boundary management, and the average “center maps” were generated using the nanmean() function, providing the likelihood profile of the label of interest as a function of the distance from the most salient position. Our recentered likelihood maps are shown on Figure 9. All three maps show a similar 2D bell-shaped activation, indicating a clear object positioning capability for all networks, retinotopic or not, when applying our “visual search” protocol (i.e., without rolling the image borders). The activation level is higher at the center (“near” the object of interest), and lower at the periphery. The likelihood values are different though, being higher on average for the Cartesian network, lower on average for the “raw” network, and more contrasted for the retinotopic network. To further quantify this contrast difference, we calculate the map difference on the second row of Figure 9, along with the log-odd ratio in the third row of Figure 9, considering one-to-one comparisons, i.e., Cartesian vs. “raw”, retinotopic vs. “raw”, and Cartesian vs. retinotopic.

Let’s analyze Figure 9. The second row shows the difference maps. The Cartesian minus “raw” map shows only positive values, while the retinotopic minus “raw” network has both positive and negative values. The retinotopic minus Cartesian map has only negative values, indicating on average a higher likelihood level in the Cartesian case. The area around the center remains close to zero though, reflecting a sharper slope towards the peripheral region in the retinotopic case. The log-odd ratio maps (third row) look quite similar at first sight to the difference maps, except in the center where the differences are more manifest. Of particular interest is the comparison of the first and second column, i.e., Cartesian vs. “raw” (first column), and Retinotopic vs. “raw” (second column). The radius of the central spot appears clearly different in the two cases, with a large central spot in the Cartesian vs. raw case, reflecting a loosy spatial discrimination improvement, and a more reduced one in the retinotopic vs. raw case, reflecting a more local and object-centric spatial discrimination improvement. Moreover, only the retinotopic network (second column) has significant peripheral depletion in comparison with the “raw” network. These observations collectively support the idea that the retinotopic network generates a heat map with enhanced contrast around the area of interest. In addition though, a focal Cartesian network also manifests good object localization capabilities, with high likelihood values around the object of interest, though being less spatially specific.

#### 3.3.2. Comparing Likelihood Maps and Intersection over UNION

**Figure 10:**
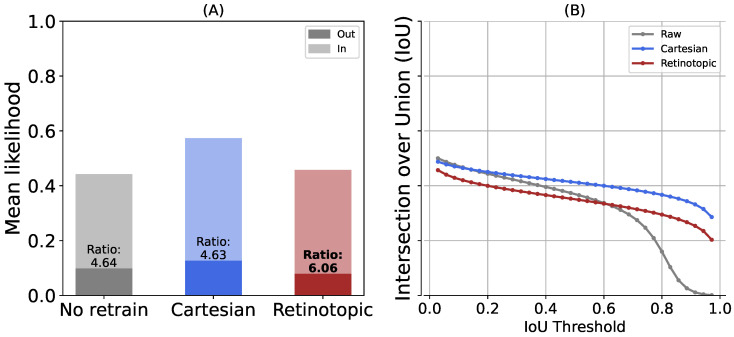
We tested the visual search protocol on the ImageNet validation dataset (50,000 images). (**A**) The mean likelihood across the point of fixation inside the bounding box (“In”) or the point of fixation outside the bounding box (“Out”) and the corresponding ratio of activation. (**B**) The intersection over union as a function of a threshold applied on the likelihood map.

To further quantify the difference between the Cartesian and retinotopic cases, we considered the localization information provided by the bounding boxes. A bounding box is defined as the rectangle of minimal surface containg one whole visual object. As such, each bounding box partitions the image into two regions, a region where the object of interest is present and a region where it is absent. Thus, given each likelihood map in the validation set, we compute the mean likelihood for the label under study *within* the bounding boxes (ground truth from the dataset) and the mean likelihood *outside* these boxes. Given the higher classification accuracies of the networks when fine-tuned on bounding boxes only the networks retrained on the “focus” dataset are used for the remainder of the study. The results are shown in Figure 10A. The figure shows a mean likelihood value obtained inside and outside the bounding boxes for the original ResNet network, our re-trained Cartesian network, and the re-trained retinotopic network. Both networks show significantly higher likelihoods when the fixation point is inside the bounding box than when it is outside the bounding box. This reflects a higher confidence in the label response. At first glance, the average likelihoods in the three conditions seem quite comparable, although the likelihood values appear slightly higher in the re-trained Cartesian case.

For a quantitative comparison, we then calculate the likelihood ratio between the area inside and outside the bounding box (see Figure 10), shown at the top of the bars: the retinotopic network has a higher likelihood ratio than the two Cartesian ones, i.e., 6.1 versus 4.6, providing quantitative evidence for a higher contrast of localization in favor of the retinotopic networks. This higher contrast is instrumental in localization tasks, as it allows for better identification of the region of interest. This effect can also be deduced from the examples shown in Figure 8: in the retinotopic case, regions of high likelihood are more sparse but still highly contrasted.

In addition, we consider in Figure 10B the Intersection over Union (IoU) metric to evaluate the agreement between the bounding boxes and the activation maps (see Section 2.6). In contrast with the “raw” network, our two networks fine-tuned on the “focus” datasets show a slower decay rate, reflecting a better fit with the ground truth. As expected from previous remarks, the IoU stays constistently higher for the cartesian network than for the retinotopic one, illustrating a tendancy for the retinotopic networks to “concentrate” likelihoods on smaller portions of the bounding box. The peak IoU is obtained for likelihood thresholds close to 0 for all networks, this could be an artifact of the measurement, since we are using the bounding box as a comparison, which encourages a larger number of positions with increased likelihood values on the map, rather than a sharp contour around the object of interest.

#### 3.3.3. Pointing Game

We have shown that the likelihood maps provide us with an indication, for each image, of the best fixation point to identify the object we are looking for. However, this spatial indication (where to place the eye) does not tell us anything about the visual content of this fixation point. To estimate this visual content more precisely, we now consider the “pointing game” metric, that is the rate of successful pointing (i.e., landing *inside* the bounding box) when choosing the highest likelihood position on the likelihood map. The bounding boxes provide us with rather coarse information about the visual content of the image, dividing it into two zones: a box within which the object is present, and a box outline where it is assumed absent. This binary information (“in” or “out”) is, however, sufficient to indicate whether the point of highest likelihood is correctly located on or in the immediate vicinity of the object of interest.

**Figure 11:**
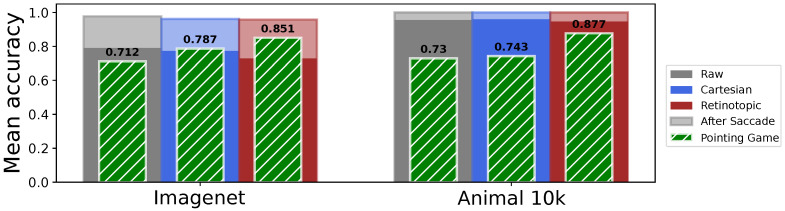
The visual search protocol is tested on the ImageNet-1K validation dataset (comprising 50,000 images) (**left**) and on the Animal 10k dataset (comprising 10,015 images) (**right**). The mean accuracy of the networks is displayed, along with the accuracy in the pointing game.

We compute for each network on the validation set the percentage of successful pointings. The results are shown on Figure 11 (green bars, left panel), on the raw, Cartesian and Retinotopic networks. The “raw” network has 71.2% success, the Cartesian has 78.74% success and the Retinotopic 85.14% success. Those rather high pointing game values reflect the fact that, on average, the bounding boxes cover a large portion of the image in the Imagenet-1k dataset. Nevertheless, the retinotopic network shows pointing scores significantly higher than those of the raw network and the Cartesian network. The position of maximal likelihood appears to be strongly correlated with the presence of the object, and this effect is notably sharper and more precise in the case of the retinotopic network.

We extend this visual search strategy by considering the classification accuracy obtained when placing the eye at the position of highest likelihood on the likelihood map, interpreted as a “saccade” toward the searched object. By doing so, the accuracy of all networks increases to about 96% (“raw” 97.7%, Cartesian 96.2%, retinotopic 95.8%, see Figure 11), providing an estimation of the *improvement* that can be expected, with respect to the baseline, when placing the eye (or camera) at its highest likelihood position (interpreted as the highest *likely* position). Consistently with our previous results, the accuracy improvement is higher in the retinotopic case, with 22.9% improvement, to be compared with 18.7% and 18.9% improvement in the “raw” and Cartesian cases respectively (see Figure 11). This strong response improvement reflects the critical role of the “direction of sight” in image classification, even in the Cartesian case, an issue little evoked in mainstream computer vision. It also suggest that the retinotopic network could be further improved by introducing such saccades during the retraining phase.

#### 3.3.4. Impact of Network Depth on Localization and Classification

We extend the analysis by considering network depths, ranging from ResNet 18 to ResNet 101. The results are shown in Table 1. We observe the same general trend across different network sizes: classification accuracy improves when the fixation point is placed at the position of highest likelihood, likelihood values are higher inside the bounding box, and the fixation point is predominantly located within the bounding box. These trends are present in both types of networks (Cartesian or Retinotopic), but they are more pronounced in the retinotopic networks. Network size primarily affects the classification rate, with lower rates observed for smaller networks. Classification rates remain slightly higher for Cartesian networks in all cases, but the improvement in classification rate based on fixation position is consistently stronger for retinotopic networks. Regarding localization (likelihood ratio and pointing game), network size does not appear to be a determining factor, with higher pointing scores and likelihood ratios observed for smaller networks, and again, a systematic advantage for retinotopic networks. In summary, Cartesian networks maintain a slight advantage in categorization, while retinotopic networks show improved performance in localization. It is noteworthy that smaller networks demonstrate better localization capabilities, challenging the conventional wisdom that performance necessarily increases with the number of layers.

### 3.4. Beyond ImageNet, the Animal-10k Dataset

ImageNet-1K provides rich semantic links that allow the construction of task-specific datasets. It has previously been demonstrated that the use of fine-tuning to re-train networks such as Vgg16 [46] allows them to be applied to different tasks using the semantic network underlying ImageNet’s labels. Furthermore, it has been shown that the probability of a trained network performing a novel task (such as categorizing an animal) can be predicted using this semantic network, which links the outputs of a pre-trained network to a library of labels. This approach has been shown to be an effective method for learning to predict the presence of animals in images [52]. We can then exploit the semantic connection underlying the ImageNet labels by using these networks to perform a categorization task on the second dataset, the Animal 10k dataset. All images were integrated in a validation dataset to test the networks. The output of each network is interpreted as “animal” if the label represented by the highest likelihood after softmax is indeed an animal. We use this prediction to calculate the accuracy of predicting an animal for all networks. Note that the accuracy is slightly biased due to the absence of distractors, it only represents the probability of finding an animal when there is actually an animal in the input. Figure 11 (right panel) compares the accuracy improvement for both networks. The general accuracy is high (around 95% in all cases) due to our simpler binary (animal/non-animal) classification task. Saccade improvement is still significant, with all networks reaching a maximal accuracy of 100% after a saccade to the maximum likelihood position for the label “animal”. The highest improvement is once again found in the retinotopic case (“raw” 4.5%, Cartesian 4.1%, retinotopic 6.3%).

**Figure 12:**
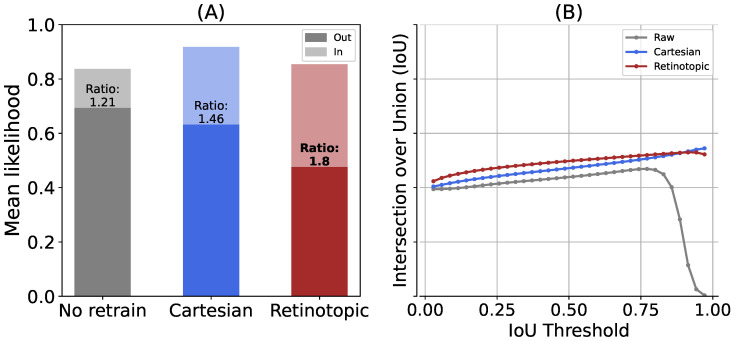
We tested the visual search protocol on the Animal 10k (comprising 10,015 images). (**A**) The mean likelihood across the point of fixation inside the bounding box (“In”) or the point of fixation outside the bounding box (“Out”) and the corresponding likelihood ratio. (**B**) The intersection over union as a function of a threshold applied on the likelihood map.

Regarding the localization capability, the In/Out likelihood ratio (Figure 12A) shows clear contrast enhancement, from 1.2 (raw network) toward 1.5 (Cartesian “focus” network) and 1.8 (Retinotopic “focus” network). The general lower contrast, when compared to ImageNet, comes again from the simpler binary (animal/non-animal) classification task, making it possible to guess the label “Animal” outside the bounding box from the background information. Looking now at the IoU metric (Figure 12B), a different trend is observed, with monotonically increasing IoU with the likelihood map threshold, reflecting a convergent matching of the likelihood map with the animal contour. The optimal threshold, close to 1, reflects a clear and sharp transition in both cases. Here, the retinotopic network provides slightly higher IoU values, except at the highest threshold case where the Cartesian network prevails. The difference between this and ImageNet lies in the fact that masks are used in the calculation instead of boxes. Last, the pointing game results (see Figure 11B, green bars) provide a clear assessment of the Cartesian/retinotopic contrast. While both the raw and Cartesian network remain quite low (73–74% success), the retinotopic framework shows much higher pointing success (87.7%) despite refined animal contour, once again suggesting a more reliable animal localization. Despite the increased diversity of poses, environments, and intra-class variability in Animal 10k compared to ImageNet-1K, our results indicate that the regions containing the target animals are effectively identified.

#### Multi-Label and Multi-Task Extension

**Figure 13:**
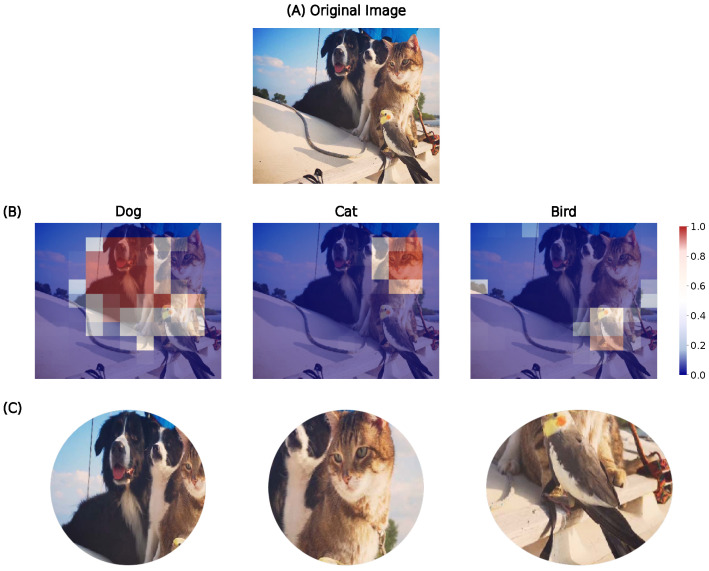
The likelihood maps presented here were generated by Resnet101 networks that were trained and tested on retinotopic space using the log-polar grid. (**A**) The original image, it is processed on a single iteration, with the likelihood maps displayed in accordance with the specific combinations of labels of interest. (**B**) Likelihood maps indicating the probability of the presence of the labels “dog”, “cat” and “bird” in the image. (**C**) The sample derived from all fixation points that yield the highest likelihood value for a label of interest.

The likelihood map can be extended along the dimension of labels. The networks perform a discriminative categorization among 1000 ImageNet labels, and the likelihood maps generated by the model vary depending on the label or class being predicted for a given input image. Specifically, the spatial distribution of high likelihood regions differs according to the object category the model is trying to identify. This suggests that the model learns to focus on different discriminative regions for different classes, providing insight into how the model’s spatial attention adapts to the visual patterns that drive each classification decision. We tested this hypothesis on an example image by generating likelihood maps for the classes “dog”, “cat”, and “bird”. As shown in Figure 13, the peak activations in these maps appear in distinct spatial regions corresponding to the locations of each respective animal in the image.

## 4. Discussion

### 4.1. Retina-Inspired Mapping Enhances the Robustness of CNNs

The first and main result of this study is to demonstrate the excellent ability of standard deep CNNs to deal with foveated retinotopic inputs, even though this mapping enforces a radical transformation of the visual inputs. ResNet networks easily adapt to these inputs and the accuracy rates achieved with retinotopic inputs are equivalent to those of the original models. This is surprising given that the networks used in this re-training process were previously trained on Cartesian images. Images with a log-polar transformation show a high degree of distortion, particularly a high compression of visual information around the fixation point and a degradation of textures in the periphery (see Figure 1). One possible hypothesis is that the degradation of texture during the frame of reference change may cause the network to rely on shape rather than texture [29]. A further study could entail a comparison of these visual processing parameters with those of human vision, thereby providing insight into their evolutionary trajectory.

In addition, the log-polar transformation has the advantage of better invariance to zooms and rotations. First, this study shows that for the original ResNet network on “regular” images, the average accuracy with a zoom or a rotation dropped sharply compared to baseline accuracy, confirming that these simple geometrical transformations misled the networks. For rotations, the decline was steepest around $160^{{}^{\circ}}$, demonstrating limited rotational invariance compared to humans [53,54]. The robustness differs slightly when studying the impact of zooms. This may be attributed to the transformation under examination, which already provides a zooming-in effect of the fovea that accentuates the information in the area surrounding the point of fixation. To test this hypothesis, we could train a retinotopic version without applying the logarithmic function to the eccentricity axis. Finally, this invariance to rotations and zooms comes at the cost of reduced invariance to translations. For images not centered on the region of interest, one would need to shift the fixation point to the region of interest, similar to eye saccades in biological vision. The effects of this trade-off can be observed in the comparison between the “regular” version of the networks and the “focus” version.

Moreover, we demonstrate an improved invariance to zoom and rotation. In the original ResNet trained on “regular” Cartesian images, accuracy declined sharply under zooms or rotations, confirming that even simple geometric transformations can mislead the network. The steepest drop occurred around $160^{{}^{\circ}}$, highlighting limited rotational invariance compared to humans [53,54]. Robustness to zoom was less affected, likely because the transformation itself magnifies foveal input relative to the periphery. Similar effects have been reported previously: Pun and Lee [55] showed that log–polar wavelet signatures enhance joint scale–rotation invariance, while Amorim et al. [56] demonstrated improved rotational robustness in natural image categorization and highlighted the positional sensitivity introduced by inhomogeneous log–polar sampling. Finally, previous research has demonstrated that improved invariance to scale variations is often associated with greater robustness to input compression [39]. This dual robustness—the ability to maintain performance across scaled inputs and compressed representations—facilitates the application of categorisation models to high-resolution images without compromising accuracy. These properties are particularly valuable in computational neuroscience, where visual processing systems must generalise across diverse input conditions. This robustness, however, comes at the cost of reduced tolerance to translations: it is sensitive to translation shifts due to the fixed fixation point of the retinotopic transform. This apparent limitation arises because the log-polar transformation assumes a fixed center of gaze, making it challenging to handle off-center objects without additional mechanisms. We see that property as an asset as the visual system in species with foveated retinotopiues are able to rapidly shift the fixation point. By exploring adaptive fixation mechanisms that dynamically adjust the center of gaze based on the input image we will preserve the benefits of foveated retinotopy.

### 4.2. From Foveation to Pre-Attentive Mechanisms

The second result of this study is the emergence of localization properties in networks re-trained with foveated inputs. Through the definition of likelihood maps, which collate the model’s output while scanning the visual scene at a limited number of fixation points, we gain insight into the specificities of retinotopic processing. This transformation provides a more focused view, thus better separating different elements of the image when focusing on specific parts. Such processing is reminiscent of pre-attentive mechanisms which allow biological vision to selectively process important zones of the visual space. As a result, retinotopic transformation provides a proxy for the measurement of saliency, particularly with respect to a set of cued labels. Overall, our findings demonstrate that foveated retinotopy introduces a meaningful trade-off: while improving robustness to rotation and zoom, it increases sensitivity to fixation shifts, which we leverage as a proxy for localization.

One hypothesis is that the foveated retinotopic mapping implemented in the log-polar transform provides an efficient prior for visual object geometry. Indeed, a main source of variability in the view of an object comes from displacements of the observer relative to it, for example as the object scales in the visual field as one approaches it, or as the object rotates with a rotation of the head. The log-polar mapping allows for a more invariant representation which explicitly implements this prior for these displacements. Additionally, imposing translation invariance of the representation establishes a prior on the possible representations allowed by the network [38]. However, it has been observed that different classes have different statistics, and the relative size of buses is on average larger than that of cats. Consequently, we expect that different foveated retinotopies may emerge in different ecological niches.

Our results indicate that the different features related to foveated retinotopy (classication robustness, localization, visual search) are tightly related. In particular, it seems that the foveated retinotopic mapping allows for a more precise localization of the category of interest compared to off-the-shelf pre-trained networks using a Cartesian representation. It also gives us insight into the features on which our networks actually rely. Such information can be compared with physiological data [57], used to design better CNNs, and ultimately enable the development of physiological tests to further explore the features needed to classify a label of interest. In particular, by focusing on the point of fixation with the highest probability in the likelihood maps instead of the center of the bounding box defined in the “focus” dataset, we could consider refining the training of the network using our retinotopic mapping in a semi-supervised fashion.

### 4.3. Perspectives and Future Work

Our results support the use of log-polar preprocessing as a useful inductive bias for improving geometric robustness and fixation-based localization in CNNs. While these findings align with principles of biological vision, further work is needed to establish broader claims about general visual robustness or biological plausibility. Our findings provide a proof of concept for the benefits of foveated retinotopy in controlled settings, particularly for centered objects. Indeed, the “*focus*” dataset introduces a central bias, as objects are centered thanks to the given bounding box. While this setting is useful for demonstrating the potential of foveated retinotopic preprocessing, it does not fully reflect real-world scenarios with off-center or multiple objects, where an efficient saccadic mechanism would be required to center the visual object. Future work should explore more diverse benchmarks to validate general-purpose robustness. Moreover, recent advances in vision-language-based perception and camouflage-aware segmentation demonstrate the potential of cross-modal priors for identifying visually ambiguous objects. For example, Zhao et al. [58] introduced a cascaded vision-language model for open-vocabulary camouflaged object segmentation, showing how integrating language and visual cues enhances robustness in challenging scenarios. While our work focuses on foveated retinotopy as a preprocessing layer, these advances highlight the broader trend of leveraging multimodal information to improve visual perception. Future work could explore integrating such cross-modal priors into our framework to further enhance robustness and interpretability.

Building on these observations, simulating human-like saccadic eye movements offers a powerful approach to probe network mechanisms. While our approach relies on a preprocessing transformation rather than architectural modifications, it offers a lightweight and interpretable alternative to attention-based or transformer-based models. Attention mechanisms, such as those in Vision Transformers (ViTs), explicitly model spatial focus but often require significant computational resources and large datasets for training [59]. Furthermore, the relationship between attention mechanisms and biological visual attention remains unclear [60]. In contrast, our log-polar preprocessing layer provides a biologically inspired inductive bias that enhances robustness to geometric transformations with minimal architectural changes. However, this approach may lack the flexibility of attention-based models in handling highly variable spatial configurations. Future work could explore hybrid models that combine the strengths of both approaches. A protocol that iteratively classifies foveated image patches could approximate natural viewing and reveal how performance varies across the visual field, as hypothesized in psychophysical experiments [61]. Comparing accuracy under different saccadic planning strategies—for example, prioritizing the most uncertain versus the most likely locations—would shed light on network attentional dynamics. This framework also enables modeling of biologically inspired saccade generation, allowing direct comparison with human visual search behavior.

Overall, implementing foveated classification with algorithmic saccades would provide a powerful method for validating existing attentional mechanisms in these networks, as well as inspiring new architectural innovations through embodied, task-driven visual attention modeling. In particular, this line of research would be especially suitable for dual-pathway models that effectively infer ego-motions [62,63]. Finally, the implementation of this robust categorization, coupled with refined localization of a label of interest and optimal saccade selection, could allow us to extend this study to more complex tasks. Visual search (i.e., the simultaneous localization and detection of a visual target) represents one such application where likelihood maps could provide the underlying pre-attentive mechanisms on which its effectiveness depends.

## Figures and Tables

**Table 1:** Analyses of the key metrics for localisation (in/out likelihood ratio and pointing game) and classification (central fixation and max likelihood (“saccade”) fixation) for different network depths.

	ResNet 18		ResNet 50		ResNet 101	
	Cartesian	Retinotopic	Cartesian	Retinotopic	Cartesian	Retinotopic
In/Out Likelihood Ratio	6.50	**9.02**	5.02	**6.40**	4.63	**6.06**
Pointing Game	83.07%	**87.40%**	80.81%	**85.13%**	78.74%	**85.14%**
Central fixation	**0.62**	0.52	**0.76**	0.71	**0.77**	0.73
With saccade	**0.92**	0.86	**0.95**	0.94	**0.96**	0.95
Accuracy increase	+30%	**+34%**	+21%	**+23%**	+19%	**+22%**

## Data Availability

All results presented here are based on data that are openly available, as described in the Section 2. For the purpose of open access, the authors have applied a CC-BY public copyright licence to any Author-Accepted Manuscript version arising from this submission. The code to reproduce all results and figures is available at https://github.com/bicv/Retinotopy (accessed on 24 March 2026).
